# Conversion of Stationary to Invasive Tumor Initiating Cells (TICs): Role of Hypoxia in Membrane Type 1-Matrix Metalloproteinase (MT1-MMP) Trafficking

**DOI:** 10.1371/journal.pone.0038403

**Published:** 2012-06-05

**Authors:** Jian Li, Stanley Zucker, Ashleigh Pulkoski-Gross, Cem Kuscu, Mihriban Karaayvaz, Jingfang Ju, Herui Yao, Erwei Song, Jian Cao

**Affiliations:** 1 Department of Medicine/Cancer Prevention, Stony Brook University, Stony Brook, New York, United States of America; 2 Department of Research, Veterans Affair Medical Center, Northport, New York, United States of America; 3 Department of Pathology, Stony Brook University, Stony Brook, New York, United States of America; 4 Department of Breast Surgery, the Memorial Hospital of Sun-Yat-Sen University, Guangzhou, China; The University of Hong Kong, Hong Kong

## Abstract

Emerging evidence has implicated the role of tumor initiating cells (TICs) in the process of cancer metastasis. The mechanism underlying the conversion of TICs from stationary to invasive remains to be characterized. In this report, we employed less invasive breast cancer TICs, SK-3rd, that displays CD44^high^/CD24^low^ with high mammosphere-forming and tumorigenic capacities, to investigate the mechanism by which stationary TICs are converted to invasive TICs. Invasive ability of SK-3rd TICs was markedly enhanced when the cells were cultured under hypoxic conditions. Given the role of membrane type 1-matrix metalloproteinase (MT1-MMP) in cancer invasion/metastasis, we explored a possible involvement of MT1-MMP in hypoxia-induced TIC invasion. Silencing of MT1-MMP by a shRNA approach resulted in diminution of hypoxia-induced cell invasion *in vitro* and metastasis *in vivo*. Under hypoxic conditions, MT1-MMP redistributed from cytoplasmic storage pools to the cell surface of TICs, which coincides with the increased cell invasion. In addition, CD44, a cancer stem-like cell marker, inversely correlated with increased cell surface MT1-MMP. Interestingly, cell surface MT1-MMP gradually disappeared when the hypoxia-treated cells were switched to normoxia, suggesting the plasticity of TICs in response to oxygen content. Furthermore, we dissected the pathways leading to upregulated MT1-MMP in cytoplasmic storage pools under normoxic conditions, by demonstrating a cascade involving Twist1-miR10b-HoxD10 leading to enhanced MT1-MMP expression in SK-3rd TICs. These observations suggest that MT1-MMP is a key molecule capable of executing conversion of stationary TICs to invasive TICs under hypoxic conditions and thereby controlling metastasis.

## Introduction

Metastasis accounts for ∼90% of all cancer deaths. Despite considerable progress in our understanding of cancer biology, leading to discovery of novel biological treatments, limitations in our understanding of invasion/metastasis has stifled development of anti-metastatic therapy.

Emerging studies have implicated cancer stem cells (CSCs)/tumor initiating cells (TICs) in the process of invasion and metastasis [1]–[3]. Based on therapeutic resistance of these cells to chemotherapy, CSCs/TICs have also been implicated in tumor recurrence. In view of the controversy surrounding the issue of “stemness”, hereafter we will designate this cell population as TICs. TICs are defined as a small subpopulation of tumor cells that display both tumor-initiating ability and the capacity to reconstitute the cellular heterogeneity of the original tumor. After initial identification in acute myelogenous leukemia [4], TICs have subsequently been isolated from many human solid tumors, including breast cancer [5]. Although there is ongoing debate [6], this subpopulation of cancer cells is acknowledged to give rise to tumors following transplantation and to have self-renewal and differentiation attributes. TICs share many of the properties of normal stem cells including dormancy (quiescence) and resistance to chemotherapy. Although the concept of tumor formation by TICs is appreciated, a fundamental gap remains in our understanding of how stationary TICs become invasive and metastatic.

Experimental and clinical evidence has demonstrated that TIC populations exhibit both migratory and invasive abilities [1] and can be readily detected in various metastatic lesions in patients with solid tumors [3], [7]. It has been proposed that under certain circumstances, TICs undergo epithelial-to-mesenchymal transition (EMT), a developmental process in which epithelial cells acquire a migratory mesenchymal phenotype. Brabletz et al. proposed that TICs acquire two phenotypes: one that is associated with cell growth and another that is migratory and characterized by transient expression of EMT-associated genes [8]. This transition can be reversed by a mesenchymal-to-epithelial (MET) transition, leading to epithelial re-differentiation, and thus enabling secondary tumor formation at metastatic sites [8].

Previously, reports have demonstrated that the low oxygen environment (hypoxia) that accompanies solid tumors induces invasiveness of cancer cells with stem-like properties [9]. Furthermore, hypoxia exerts a physiological selection pressure that leads to enhanced tumor invasion and metastasis [10].

Considerable evidence indicates that cancer cells and TICs require proteases for their invasive behavior [11]. A key cell surface anchored protease, membrane type 1-matrix metalloproteinase (MT1-MMP), plays a critical role in digesting basement membrane and extracellular matrices (ECM) and in cancer cell migration [12], hence promoting cancer invasion and metastasis. We have previously demonstrated a role for MT1-MMP in EMT [13]. In the current study, we employed a human breast cancer TIC line, SK-3rd, to study the function of MT1-MMP in the conversion of TICs from stationary to invasive cells. This TIC line was isolated by sequential *in vivo* passage of SK-BR3 human breast cancer cells in immuno-deficient mice undergoing treatment with chemotherapy [14]. We demonstrated that hypoxia induces MT1-MMP trafficking from cytoplasmic storage pools to the plasma membrane, promoting TICs invasion.

## Results

### Hypoxia Promotes TIC Invasion

**Figure 1:**
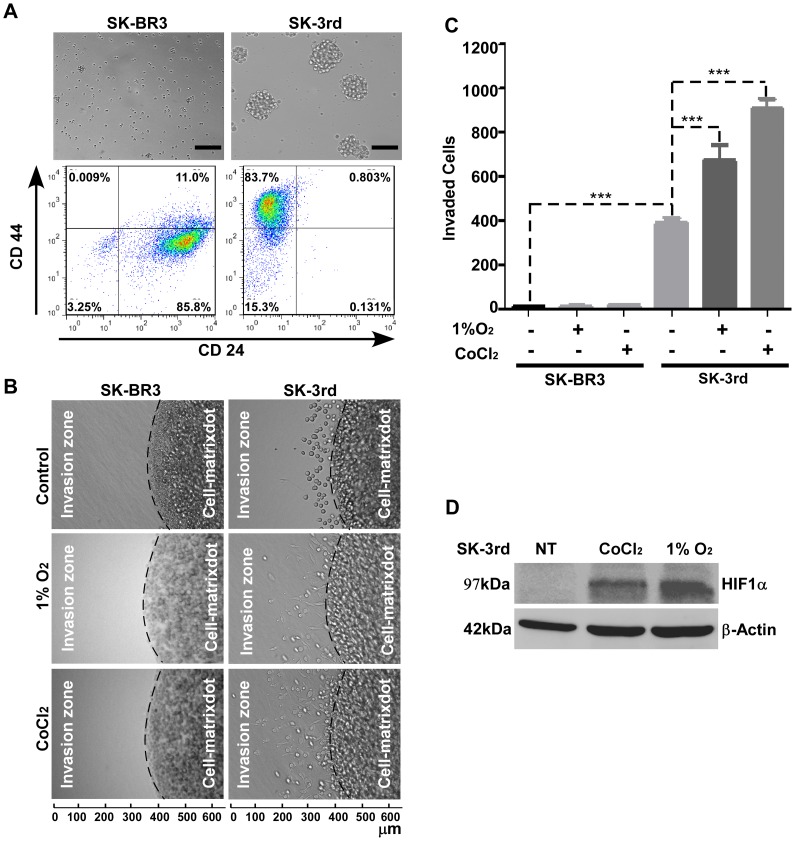
Hypoxia Enhances TIC Invasion. A) Characterization of SK-3rd TICs: SK-3rd and SK-BR3 cells were cultured on ultra-low attachment culture dishes for 3 days and mammosphere formation was examined by microscopy (Upper panel). Bar, 100 µm. These cells were collected and incubated with APC-conjugated anti-CD44 antibodies and PE-conjugated anti-CD24 antibodies followed by flow cytometry analysis (Lower panel). B–C) Determination of invasive ability of SK-3rd TICs: SK-BR3 and SK-3rd cells were pretreated with or without CoCl_2_ (250 µM) or cultured under hypoxia (1% O_2_) for 24 hours followed by examination of invasive ability of the cells using the 3-D invasion assay (B). After 24 hours, the cells were fixed and invaded cells in six different fields in the invasion zone were microscopically counted (C). D) Evaluation of hypoxic conditions by examining HIF-1α in SK-3rd cells: Total cell lysate from SK-3rd TICs pretreated with or without CoCl_2_ (250 µM) or under 1% O_2_ for 24 hours were examined by Western blotting using anti-HIF-1α antibody. NT refers to cells without pre-hypoxia culture.

The mechanism by which stationary TICs convert to their metastatic counterpart and then return to stationary status at the metastatic site remains to be characterized. To study this phenomenon, we employed a previously established and well characterized TIC line, SK-3rd [14]. These cells display a cancer stem-like cell phenotype including self-renewal (exhibited as an enhanced mammospehere formation), cell surface markers for breast TICs (CD44^high^/CD24^low^) (Fig. 1A), and enhanced tumorigenicity (Table 1). In agreement with the initial report of Yu *et al*. [14], we noted that SK-3rd cells expressing GFP display enhanced lung metastasis as compared to parental SK-BR3 cells expressing GFP (Table 1).

**Table 1:** In Vivo Tumorigenicity and Spontaneous Metastasis Assays.

		Number of cells			
		4×10^3^		4×10^4^	
Cell lines	DNA	Tumor	Lung Metastasis	Tumor	Lung Metastasis
SK-BR3	GFP	n.d.	n.d.	0/5	0/5
	MT1- GFP	n.d.	n.d.	0/5	0/5
SK-3rd	GFP	5/5	3/5	n.d.	n.d.
	MT1- GFP	5/5	4/5	n.d.	n.d.
	shRNA-Luc	5/5	3/5	n.d.	n.d.
	shRNAMT1	5/5	1/5	n.d.	n.d.

“n.d.” = not done.

It has been established that metastasis requires cancer cells with enhanced invasive ability. To dissect the metastatic process of SK-3rd TICs observed *in vivo*, a three-dimensional (3-D) invasion assay was employed. Mammosphere-forming SK-3rd TICs as well as parental SK-BR3 cells were assembled in type I collagen gel and cultured under normoxic conditions (21% O_2_) for 24 hours. Unexpectedly, the invasive ability of SK-3rd TICs was only moderately increased as compared to parental SK-BR3 cells, which lacked invasive ability (Fig. 1B & C). This result is inconsistent with the *in vivo* studies (Table 1) [14]. Given the evidence that SK-3rd TICs develop into relatively rapid growing and metastatic tumors *in vivo* and rapid growing solid tumors often contain regions lacking sufficient oxygenation [15], we hypothesized that hypoxia may be responsible for SK-3rd cell invasion and metastasis. To test the effect of hypoxia on SK-3rd TIC invasion, we employed a hypoxia-mimicking chemical agent, CoCl_2_, to recapitulate the effects of hypoxia [16]. The effect of hypoxia on TIC invasion was also assessed under 1% O_2_ atmosphere. Hypoxic conditions were confirmed by Western blotting using antibody against hypoxia-inducible factor-1α (HIF-1α), an intrinsic marker of hypoxia [17] (Fig. 1D). SK-3rd and SK-BR3 cells pretreated with CoCl_2_ or cultured under hypoxia (1% O_2_) were examined for their invasive abilities in the 3-D invasion assay. Significantly increased cell invasion into surrounding type I collagen was observed in SK-3rd TICs treated with CoCl_2_ as compared to vehicle control. Similar result was observed when the cells were cultured under hypoxic conditions (1%O_2_). In contrast, parental SK-BR3 cells either treated with CoCl_2_ or cultured under hypoxic conditions did not display enhanced cell invasion (Fig. 1B & C). These data suggest that the invasive ability of SK-3rd TICs is regulated by hypoxia.

### Relocation of MT1-MMP from Cytoplasmic Pools to the Cell Surface Enhances TIC Invasion

**Figure 2:**
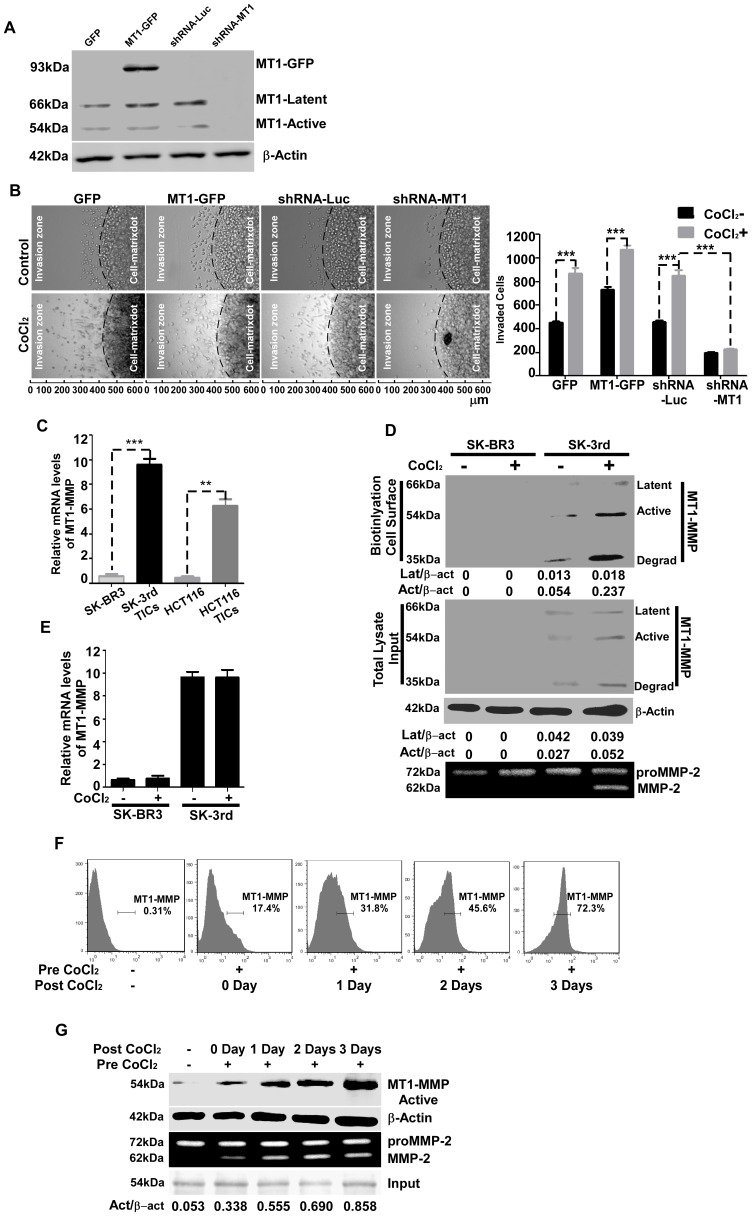
Hypoxia induces intracellular MT1-MMP trafficking to the cell surface, resulting in enhanced invasiveness of SK-3rd cells. A) Determination of MT1-MMP expression in SK-3rd cells: Total cell lysates from SK-3rd cells either overexpressing of MT1-GFP or silencing of endogenous MT1-MMP were analyzed by Western blotting using anti-MT1-MMP antibodies. β-Actin was used as a loading control. B) Examination of the role of MT1-MMP in SK-3rd TIC invasion: SK-3rd TICs with overexpression or downregulation of MT1-MMP as indicated were pretreated with or without CoCl_2_ (250 µM) for 24 hours followed by evaluation of cell invasive ability in the 3-D invasion assay (Left panel). The invaded cells were microscopically counted (Right panel). Hypoxia promotes SK-3rd TIC invasion and this process requires expression of MT1-MMP in the cells. C) Expression of MT1-MMP in TICs: Total RNAs from breast cancer cells (SK-BR3 parental cells and SK-3rd TICs) and colon cancer cells (HCT116 parental cells and HCT116 TICs) were examined for the expression of MT1-MMP using a real time RT-PCR approach. Housekeeping genes (GAPDH and HPRT) were used to normalize MT1-MMP mRNA in all samples. D) Determination of functional MT1-MMP in SK-3rd TICs: Top panel: Cell surface MT1-MMP in SK-BR3 and SK-3rd cells treated with or without CoCl_2_ for 24 hours were biotinylated and precipitated with streptavidin-beads followed by Western blotting using anti-MT1-MMP antibodies. Densitometry analysis was performed and ratios between MT1-MMP latent and active forms over β-actin were given. Middle panel: An aliquot of total cell lysates of cell surface biotinylated SK-BR3 and SK-3rd cells were examined by Western blotting using anti-MT1-MMP antibodies. β-actin was used as a loading control. Densitometry analysis was performed and ratios between MT1-MMP latent and active forms over β-actin were given. Bottom panel: The conditioned medium in the presence of recombinant proMMP-2 was examined by gelatin zymogram to determine functional MT1-MMP. E) Effect of CoCl_2_ on MT1-MMP expression: SK-BR3 and SK-3rd cells treated with or without CoCl_2_ (250 µM) for 24 hours were examined by real time RT-PCR using MT1-MMP specific primers and housekeeping genes for normalization. F–G) Correlation of cell surface expression of MT1-MMP with proMMP-2 activation: SK-3rd TICs pretreated with CoCl_2_ (Pre CoCl_2_; 250 µM) for 24 hours were switched to complete medium in the absence of CoCl_2_ (Post CoCl_2_) for indicated dates followed by flow cytometry analysis using anti-MT1-MMP antibody (F). The cells were also examined by cell surface biotinylation experiment using antibodies for MT1-MMP (G, Upper panel) and β-actin (G, loading control, Middle panel), and gelatin zymogram (G, Lower panel) and input of MT1-MMP (Bottom panel). Densitometry analysis was performed and ratios between MT1-MMP active form over β-actin was given.

A previously fine-tuned analysis of proteases with collagenase activity suggested that only MT1-MMP confers the focal collagenolytic activity necessary to support the tissue-invasive cell phenotype [18]. To examine the role of MT1-MMP in hypoxia-induced TIC invasion in 3-D type I collagen gels, both loss- and gain-of-function assays were performed using our previously generated MT1-MMP-GFP chimeric cDNA (MT1-GFP) [12] and shRNAs against MT1-MMP [13]. Overexpressing or silencing of MT1-MMP in SK-3rd TICs were characterized by Western blotting using anti-MT1-MMP antibody (Fig. 2A). Silencing of MT1-MMP in SK-3rd TICs resulted in a defect in cell invasion in the presence of CoCl_2_, whereas overexpression of MT1-MMP in SK-3rd TICs significantly enhanced cell invasion (Fig. 2B). These loss- and gain-of-function assays led us to further analyze endogenous MT1-MMP expression in SK-3rd using biochemical approaches. We first examined basal levels of MT1-MMP expression in SK-3rd and parental SK-BR3 cells by quantitative real time RT-PCR. MT1-MMP was up-regulated more than ten-fold in SK-3rd cells compared to parental SK-BR3 cells (Fig. 2C). Similar results were found in TICs derived from human HT116 colon cancer compared to its parental cells (Fig. 2C). This increase of MT1-MMP mRNA correlated with protein expression levels as examined in total cell lysates by Western blotting (Fig. 2D, Middle panel, non CoCl_2_-treated SK-BR3 and SK-3rd). In agreement with previous report [19], [20], three forms of MT1-MMP were detected in the total cell lysates. Since hypoxia was found to increase TIC invasion (Fig. 1B), we asked if increased invasive ability of SK-3rd under hypoxia was due to upregulated MT1-MMP expression. Surprisingly, hypoxia did not change the mRNA level of MT1-MMP in SK-3rd TICs in the presence CoCl_2_ (Fig. 2E), suggesting that hypoxia plays a minimal role in regulation of MT1-MMP gene expression.

It has been demonstrated that plasma membrane distribution of MT1-MMP is required for its functionality [21]. To determine if hypoxia-enhanced cell invasion is due to MT1-MMP cell surface redistribution, a cell surface biotinylation assay followed by Western blotting was performed. As demonstrated in Figure 2D, the 54 kDa active form, as well as the degradation product of MT1-MMP was increased on the cell surface of SK-3rd TICs after 24 hours of CoCl_2_ treatment. Increased cell surface MT1-MMP correlated with proMMP-2 activation (Fig. 2D, Lower panel), indicative of functional MT1-MMP. This observation was not seen in MT1-MMP-silenced SK-3rd TICs (data not shown).

To examine the dynamic distribution of cell surface MT1-MMP in response to CoCl_2_ treatment, SK-3rd TICs were treated with CoCl_2_ followed by flow cytometry analysis. One to three days post CoCl_2_-treatment, 1×10^4^ cells were used to gate the proportion of positive stained cells at each time point and the percentage of positive cells was determined. Dead cells were excluded by gating out propidium iodide (PI)-positive cells. The population of SK-3rd cells expressing cell surface MT1-MMP gradually increased under CoCl_2_-treatment (Fig. 2F). To confirm this observation, a cell surface biotinylation assay was performed. In agreement with the flow cytometry analysis, cell surface biotinylation studies demonstrated that active MT1-MMP (54 kDa) at the cell surface was markedly and progressively increased over a three day period (Fig. 2G, Upper panel). This increase was correlated with functional MT1-MMP, as examined by gelatin zymography (Fig. 2G, Lower panel). These observations suggest that MT1-MMP is capable of converting less invasive, stationary TICs into invasive cancer cells by promoting MT1-MMP relocation to plasma membrane.

### MT1-MMP Induces Dynamic Change of Cell Surface CD44 and Mammosphere-forming Ability under Hypoxic Conditions

**Figure 3:**
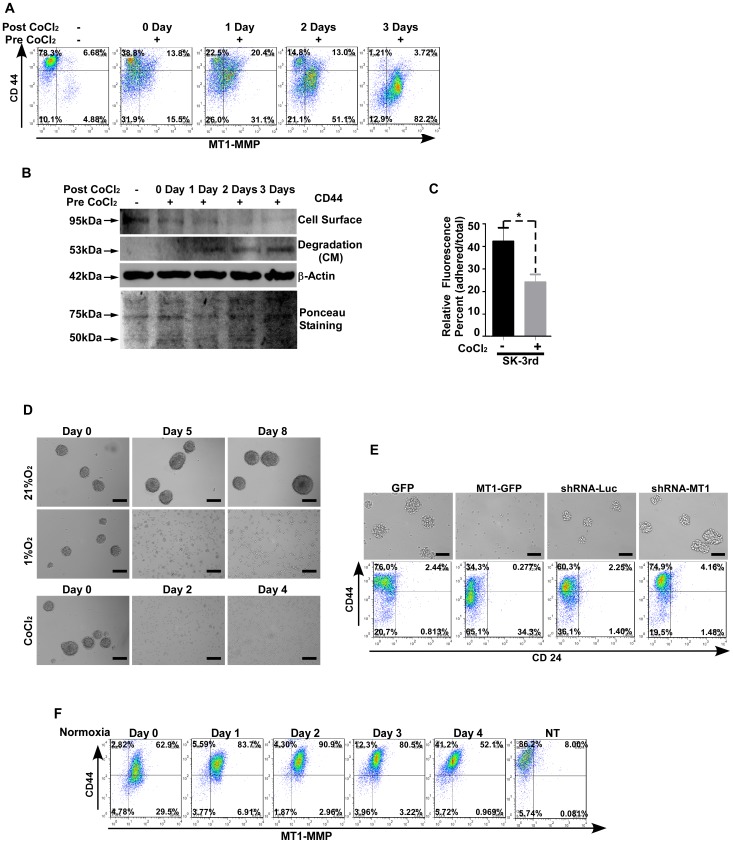
Effect of hypoxia-induced cell surface MT1-MMP on cell surface CD44 and mammosphere formation. A–B) Cell surface CD44 inversely correlates with hypoxia-induced cell surface MT1-MMP: SK-3rd TICs were cultured in the presence of CoCl_2_ (Pre CoCl_2_; 250 µM) for 24 hours and then switched to CoCl_2_-free medium (Post CoCl_2_) for indicated times. Flow cytometry analysis employing APC-anti-CD44 antibodies and anti-MT1-MMP antibodies in conjunction with FITC-labeled secondary antibodies (A). Cell surface proteins were biotinylated and examined for CD44 appearance (B, Upper panel). Shed CD44 in the conditioned medium was examined by Western blotting using anti-CD44 antibodies (B, Middle panel). β-actin was used as a loading control (B, Lower panel). Ponceau staining of the membrane with samples from the conditioned medium was employed as a loading control. C) Effect on cell adhesion by hypoxia using a hyaluronic acid adhesion assay: SK-3rd TICs treated with or without CoCl_2_ (250 µM) for 24 hours were examined for their ability to adhere to hyaluronic acid-coated 96-well tissue culture plates. The adherent cells were enumerated based on calcein staining. D) Dissociation of mammospheres by low oxygen (Upper panel) or CoCl_2_ treatment: Mammospheres from SK-3rd TICs were cultured under either 21% O_2_ or 1% O_2_ or treated with CoCl_2_ (250 µM) for indicated times. The cells were microscopically examined daily. Bar, 100 µm. E) Effect of MT1-MMP on mammosphere forming ability and cell surface CD44 expression: SK-3rd TICs were infected with retrovirus containing cDNAs for GFP or MT1- GFP and shRNAs against luciferase (shRNA-Luc) or MT1-MMP (shRNA-MT1). The cells were cultured in ultra-low attachment culture dishes for their mammosphere forming abilities (Upper panel) and cell surface expression of CD44 and CD24 were examined by flow cytometry using corresponding antibodies (Lower panel). F) Reversal of hypoxia-induced MT1-MMP under normoxic conditions: SK-3rd TICs were cultured under 1% O_2_ for 24 hours and then switched to normoxic conditions for indicated time. Flow cytometry analysis was performed using anti-CD44 and anti-MT1-MMP antibodies. NT refers to non-hypoxia-cultured cells.

CD44 has been implicated as one of the TIC markers in hematopoietic and epithelial malignancies [22], [23]. Studies have shown that CD44 is implicated in several cancer processes including cell growth, adhesion, invasion, and metastasis [24]. To address the effect of enhanced cell surface MT1-MMP on CD44 appearance in TICs under hypoxic conditions, SK-3rd cells treated with CoCl_2_ were analyzed by flow cytometry using CD44 (APC-labeled) and MT1-MMP (FITC-labeled) antibodies. Gradually increased cell surface MT1-MMP was found to correlate with decreased cell surface CD44 (Fig. 3A). Decreased cell surface CD44 was not due to an effect of hypoxia on CD44 synthesis, as evidenced by real time RT-PCR using CD44 specific primers (data not shown), suggesting that increased MT1-MMP under hypoxia results in shedding of cell surface CD44. To fortify this observation, conditioned medium and cell surface proteins were examined using biochemical approaches. Treatment of SK-3rd TICs with CoCl_2_ resulted in diminished cell surface CD44 (95 kDa), as examined by a cell surface biotinylation assay. This decrease was accompanied by increased soluble CD44 (54 kDa) in the conditioned medium, as examined by Western blotting using anti-CD44 antibodies (Fig. 3B). To correlate shedding of CD44 with the loss of function, a hyaluronic acid adhesion assay was performed to determine functionality of CD44 [25]. SK-3rd TICs pretreated with CoCl_2_ for 24 hours were examined for adhesive ability to hyaluronic acid-coated plates. Treatment of SK-3rd cells with CoCl_2_ resulted in diminished cell adherence to hyaluronic acid-coated plates, consistent with shedding of cell surface CD44 (Fig. 3C).

Mammosphere formation is another intrinsic property of TICs. To determine the effect of shedding of CD44 by MT1-MMP on maintaining mammospheres of TICs, SK-3rd TICs were cultured in ultra-low adherence plates in the hypoxic (1% O_2_) or normoxic (21% O_2_) conditions for indicated times. Mammoshperes cultured under hypoxic conditions gradually dissociated into single, isolated cells (Fig. 3D, Upper panel). In contrast, mammospheres in normoxic condition continued to expand. A similar result was obtained in mammospheres derived from SK-3rd cells treated with CoCl_2_ (Fig. 3D, Lower panel).

To confirm this observation, both gain- and loss-of-function assays were performed. SK-3rd TICs were stably infected with a retrovirus containing MT1-GFP chimeric cDNAs as well as GFP control cDNAs. Overexpression of MT1-GFP in SK-3rd TICs resulted in a decrease of CD44 as compared to GFP control examined by flow cytometry (Fig. 3E, Lower panel). Furthermore, overexpressing MT1-GFP in SK-3rd TICs resulted in loss of mammosphere-forming ability (Fig. 3E, Upper panel). In the loss-of-function assay, silencing of endogenous MT1-MMP (shRNA-MT1) in SK-3rd cells increased cell surface CD44 as compared to luciferase shRNA control. In agreement with increased CD44, silencing of MT1-MMP enhanced mammosphere-forming ability (Fig. 3E, Lower panel). However, both gain- and loss-of-function approaches had no notable effect on tumorigenicity after orthotopically injecting 4×10^3^ tumor cells into mice (Table 1). Expression of CD24 was not affected in both gain- and loss-of-function assays.

During metastasis, the oxygen environment surrounding cancer cells undergoes dynamic changes. To determine if fluctuation of oxygen tension affects MT1-MMP cell surface expression and the subsequent change in cell surface CD44, SK-3rd TICs cultured under hypoxia (1% O_2_) for 4 days were switched to normoxic conditions for 1 to 4 days followed by flow cytometry analysis using anti-MT1-MMP and anti-CD44 antibodies. Return to normoxia was accompanied by a gradual decrease in display of cell surface MT1-MMP and increase in cell surface CD44 (Fig. 3F). These data suggest dynamic changes of properties of cancer stem-like cells during cancer progression.

### MT1-MMP Initiates Tumor Invasion and Metastasis *in vivo*

**Figure 4:**
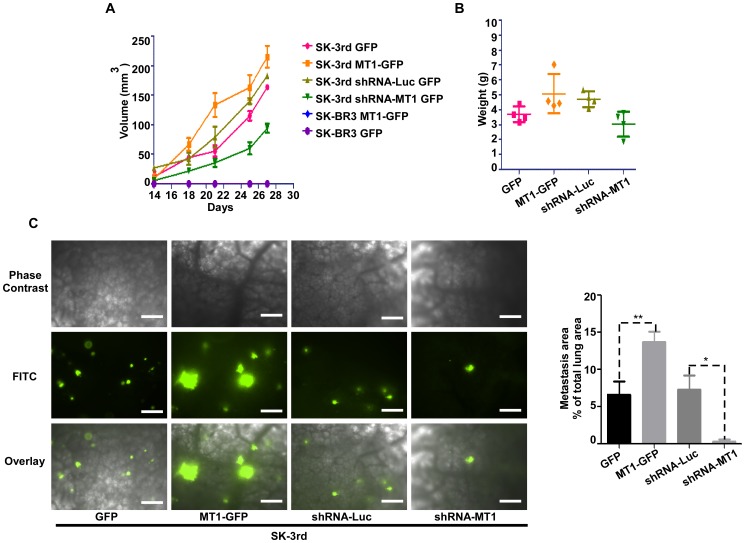
MT1-MMP is required for metastasis of SK-3rd TICs. SK-3rd TICs expressing exogenous genes for either upregulation or downregulation of MT1-MMP as indicated were injected into immunodeficient mice and tumor growth was monitored (A). After four weeks, the experiments were terminated and entire tumor was harvested and weighed (B). The lungs were examined under phase contrast (C, Lift upper panel) and fluorescent (Right lower panel) microscopy. Metastasized tumors were identified based on GFP auto-fluorescence and measured using Nikon NIS Elements. Quantification of total lung metastasis burden in mice bearing tumor cells as indicated was determined based on percentage of metastasized tumors (area) over lungs (area) under the same field (C, Right panel). Bar, 100 µm.

A previous study demonstrated that SK-3rd TICs display distant metastasis in immunodeficient mice [14]. To determine the role of MT1-MMP in SK-3rd TIC invasion and metastasis, both loss-of-function (silencing endogenous MT1-MMP [shRNA-MT1] in SK-3rd TICs) and gain-of-function (overexpressing of MT1-GFP in SK-3rd TICs) were employed. These TICs (4×10^3^) were then injected into mammary fat pads of NCR nude mice. Parental SK-BR3 cells (4×10^4^) expressing GFP or MT1-GFP were used as controls. The experiment was terminated after 4 weeks when the primary tumor size reached 1.2 cm in diameter in mice bearing MT1-MMP-GFP transfected cells. Except for SK-BR3 cells, all mice displayed visible mammary tumors within 2 weeks after tumor cell injections, indicating that MT1-MMP had minimal effect on ultimate incidence of tumor formation. However, SK-3rd cells overexpressing MT1-GFP displayed more rapid tumor growth than control cells, whereas MT1-MMP silenced tumors had a slower growth rate than control SK-3rd cells expressing luciferase shRNA (p<0.05) (Fig. 4A, B). These observations indicate that MT1-MMP expression in TICs enhances tumor growth *in vivo*.

Metastases from primary tumors were quantified by assessing GFP fluorescence in lung tissue. Mice injected with MT1-GFP overexpressing SK-3rd cells displayed larger volume pulmonary metastasis than GFP overexpressing SK-3rd cells (Fig. 4C). In contrast, diminished lung metastasis was found in mice injected with MT1-MMP-silenced SK-3rd cells, suggesting the role of MT1-MMP in TIC metastasis.

### Breast TICs Possess Properties of EMT

**Figure 5:**
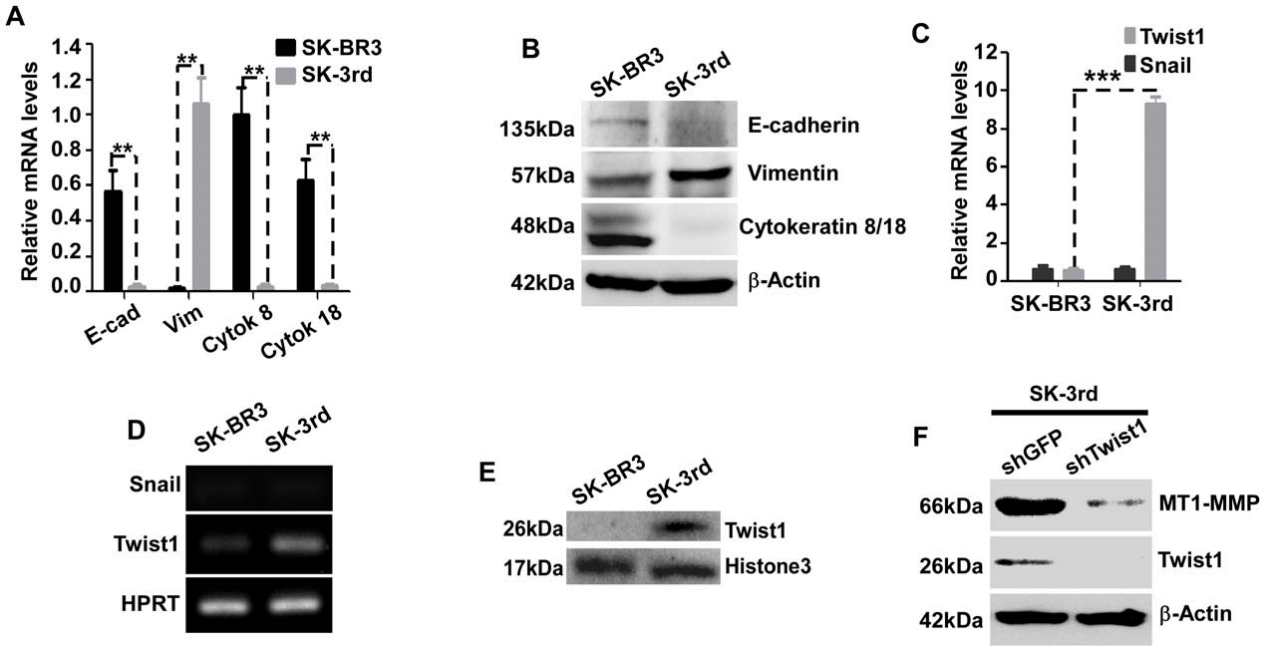
SK-3rd TICs possess properties of EMT. A–B) Differential expression of EMT markers in SK-3rd TICs: SK-BR3 and mammosphere forming SK-3rd cells were examined by real time RT-PCR (A) and Western blotting (B) for expression of EMT markers. E-cad: E-cadherin; Vim: Vimentin; Cytok: cytokeratin. C–E) Upregulation of Twist-1 in SK-3rd TICs: The total RNA of SK-BR3 and SK-3rd cells was examined for expression of Twist-1 and Snail using real time RT-PCR (C). Housekeeping genes were used to normalize Twist-1 and Snail mRNAs in all samples. At the end of real time RT-PCR, the samples were examined in a 1.2% agarose gel (D). The cell lysates from nuclear fractions were examined by Western blotting using anti-Twist-1 antibodies (E, Upper panel). Histone 3 was used as a loading control for nuclear preparation. F) Effect of Twist-1 on MT1-MMP expression: SK-3rd TICs silenced for Twist-1 or GFP control were examined by Western blotting using antibodies as indicated for expression of MT1-MMP and Twist-1. β-actin was used as a loading control.

Previous studies have demonstrated that the transcription factors Twist-1 and Snail play essential roles in tumor metastasis [26], [27]. Transfection of Twist-1 or Snail cDNAs into human mammary epithelial cells (HMLE) resulted in conversion of epithelial cells to a stem-like phenotype accompanied by EMT [28]. To address the correlation between “stemness” and EMT in our TIC model, we examined EMT markers in cultured cancer cells employing real time RT-PCR and Western blot analysis. As compared to SK-BR3 cells, SK-3rd cells displayed downregulated expression of epithelial markers, E-cadherin, cytokeratins-8 and -18, and upregulated expression of the mesenchymal marker, vimentin (Fig. 5A, B). In addition, mRNA and protein expression of Twist-1, but not Snail, was increased in SK-3rd cells as compared to SK-BR3 (Fig. 5C, D, & E). We have previously demonstrated that MT1-MMP plays an important role in epithelial cancer cell EMT [13]. To probe if Twist-1 is an upstream regulator of MT1-MMP and subsequently controls EMT, we silenced Twist-1 by shRNA in SK-3rd cells. A significant decrease of MT1-MMP expression was detected by Western blotting analysis (Fig. 5F), confirming the role of Twist-1 in regulation of MT1-MMP.

### Regulatory Mechanism of MT1-MMP in SK-3rd TICs

**Figure 6:**
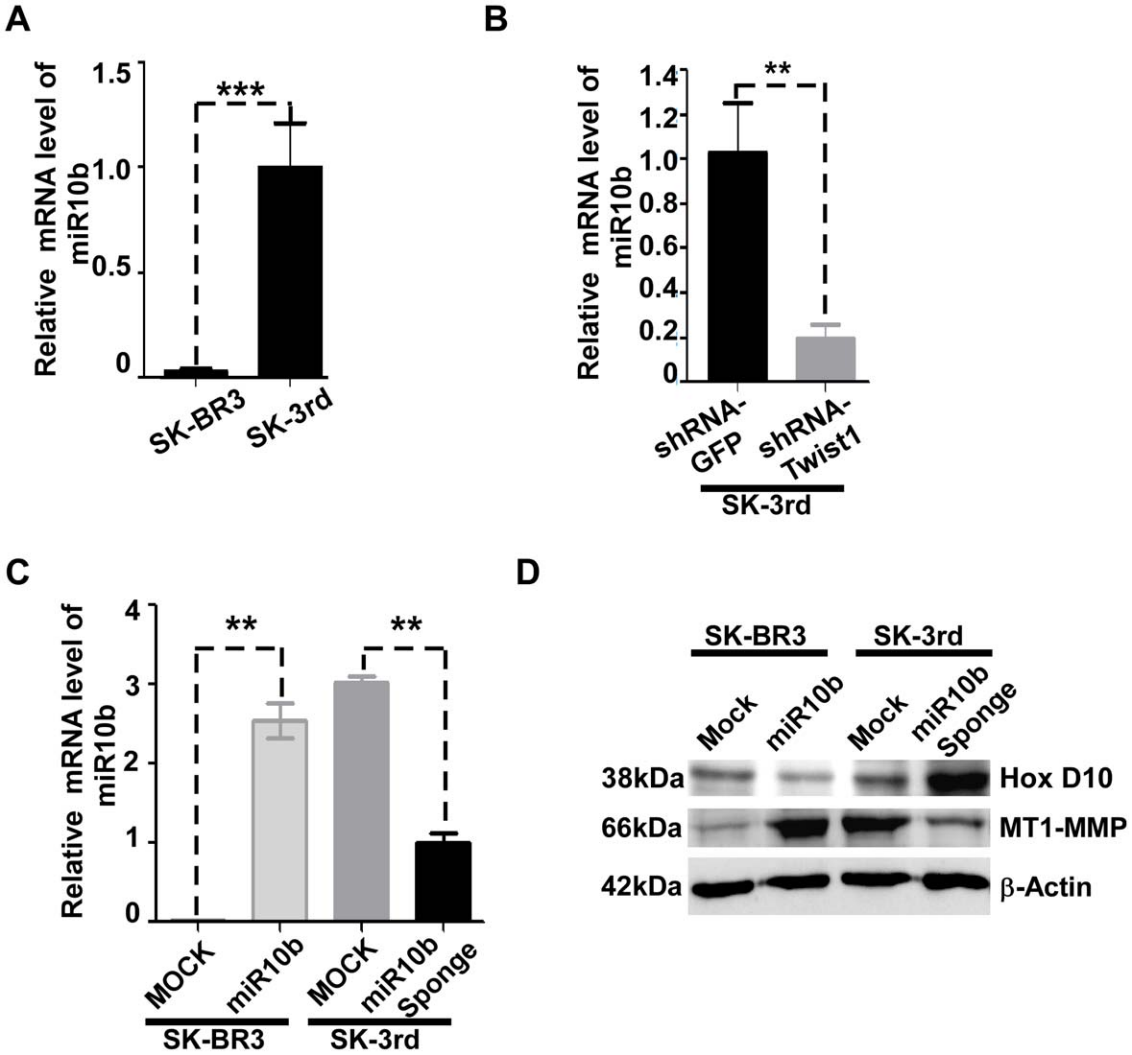
miR10b regulates MT1-MMP through HoxD10. A–C) Expression of miR10b in SK-3rd TICs or Twist-1 silenced-SK-3rd TICs: Total RNA was examined for expression of miR10b in cells as indicated by real time RT-PCR. Housekeeping gene (RNU6B) were used to normalize miR10b in all samples. D) Effect of miR10b on HoxD10 and MT1-MMP expression: Total cell lysates from cells as indicated were examined by Western blotting using anti-HoxD10 and MT1-MMP antibodies, respectively. β-actin was used as a loading control.

Although induction of MT1-MMP expression by growth factors and cytokines has been reported [29], a detailed regulatory mechanism of MT1-MMP expression remains elusive. Recent reports have suggested that a cascade of miR10b-HoxD10 may regulate MT1-MMP expression, although it is unclear how HoxD10 controls MT1-MMP expression [30], [31]. To examine expression level of miR10b in SK-3rd TICs, real time RT-PCR using specific primers for miR10b was employed. miR10b was found only in SK-3rd cells, as compared to parental SK-BR3 cells (Fig. 6A). This observation is in agreement with a previous report [30], [31] that expression of Twist-1 resulted in elevated miR10b (Fig. 6B).

HoxD10 has been reported to be a suppressor of cell invasion and migration [32] and a direct target of miR10b [30]. Following apparent binding to the promoter region of MT1-MMP, HoxD10 has been proposed to suppress MT1-MMP expression [31]. To provide experimental evidence for this hypothesis, we silenced miR10b using a vector encoding anti-sense of miR10b, designated as miR10b sponge [30] in SK-3rd cells. Decreased miR10b in SK-3rd TICs by miR10b sponge was confirmed by real time RT-PCR (Fig. 6C). Silencing of miR10b resulted in increased HoxD10 expression in SK-3rd TICs examined by Western blotting analysis (Fig. 6D upper panel). This increase of HoxD10 led to downregulated MT1-MMP protein expression in the cells (Fig. 6D). To further address the effect of miR10b-HoxD10 cascade on MT1-MMP expression, miR10b was overexpressed in parental SK-BR3 cells. Overexpressing of miR10b in SK-BR3 cells resulted in diminished HoxD10 and increased MT1-MMP protein expression (Fig. 6D).

Taken together, our data demonstrate a cascade of Twist1-miR10b-HoxD10 in control of MT1-MMP expression. Furthermore, these data provide evidence for a mechanism of regulating MT1-MMP expression in less invasive SK-3rd TICs under normoxic conditions.

## Discussion

In this study, we demonstrated that MT1-MMP is generally expressed and stored in a non-functional cytoplasmic pool in TICs. This upregulation of MT1-MMP is transcriptionally controlled by a cascade involving Twist1-miR10b-HoxD10. Under hypoxic conditions, which often occur in fast growing tumors, MT1-MMP rapidly trafficks to the plasma membrane to execute cell escape actions away from the hypoxic environment. This process results in enhanced cell migration, invasion, and metastasis. Our data demonstrate for the first time that less invasive and stationary cancer stem-like cells can become more motile and invasive under hypoxic conditions.

Due to technical limitations of maintaining TIC status in culture, TICs have been difficult to study; this difficulty has hampered anti-TIC drug discovery. Recent TIC research efforts have advanced our capability for generating stable TIC lines [14], [28]. The SK-3rd TIC line used in this study meets current definitions for TICs (cancer stem-like cells). This includes: 1) mammosphere formation, which is a measure of self-renewal for stem-like cells; 2) cell surface markers of breast TICs (CD44^high/^CD24^low^); and 3) *in vivo* tumorigenic capability requiring relatively low number of cells. Although SK-3rd TICs display invasive ability in type I collagen gel, this property of SK-3rd TICs is minimal as compared to invasive breast cancer cell lines, such as MDA-MB-231 (data not shown). In addition, the invasive ability of SK-3rd TICs is markedly enhanced under hypoxic conditions. Given these observations, SK-3rd TICs are considered less invasive, stationary TICs.

Emerging evidence has indicated that the hypoxia that accompanies solid tumors induces invasiveness of cancer cells with stem-like properties [9], [33]. Furthermore, hypoxia exerts a pathophysiological selection pressure that leads to enhanced tumor invasion and metastasis [10], [34]. In the current study, two experimental models were used to mimic a hypoxic environment: a hypoxia chamber with 1% O_2_ and a well-known hypoxia mimetic agent, CoCl_2_. Both models work via stabilizing HIF-1α. This has been confirmed by Western blotting using anti-HIF-1α antibody in our working system (Fig.1D). Although similar results were obtained using these two models, we found that the effect of CoCl_2_ on MT1-MMP-induction lasts longer compared to that induced by the hypoxia chamber, when cells were switched from hypoxic to normoxic conditions (data not shown).

Despite continuous progress in identifying potential therapeutic targets for prevention of metastasis, the contribution and timing of key regulators remains unclear [35]. Regardless of which molecules are primarily responsible for initiating cancer dissemination, mounting evidence has demonstrated that cancer cells and TICs require proteases for their invasive behavior. We focused on a key cell surface-anchored protease, MT1-MMP, to characterize the mechanism of hypoxia-induced TIC invasion in type I collagen gel. This is based on a recent report demonstrating that MT1-MMP is the only protease responsible for the focal collagenolytic activity necessary to enhance tumor cell invasion [36]. Our data support that MT1-MMP plays a key role in enhancing TIC invasion in type I collagen gel. Since MT1-MMP is highly expressed in most human cancers [37], utilization of cell surface MT1-MMP as a cancer-homing molecule for delivering potent cytotoxic drugs, particularly to invasive TICs, provides a viable approach to enhance cancer cell target selectivity and specificity.

Compared to parental breast cancer SK-BR3 cells, expression level of MT1-MMP in SK-3rd TICs is elevated. Although MT1-MMP has been identified for more than a decade [38], the regulatory mechanism of MT1-MMP remains to be characterized. Growth factors and cytokines have been reported to induce MT1-MMP expression [37]. Our studies demonstrate that Twist-1 is upregulated in SK-3rd TICs, which exhibit an EMT phenotype (Fig. 5A, B). Twist-1 has previously been reported to play an essential role in cancer cell EMT and subsequently tumor invasion and metastasis [27]. We previously reported that MT1-MMP plays an important role in prostate cancer cell EMT [13]. A possible link between Twist-1 and MT1-MMP in EMT is proposed. Indeed, silencing of Twist-1 gene expression in SK-3rd TICs significantly reduced MT1-MMP protein expression (Fig. 5F). This is in part due to the upregulated Twist-1 in SK-3rd TICs leading to miR10b production, which then suppresses a MT1-MMP transcriptional suppressor, HoxD10, resulting in upregulated MT1-MMP in TICs cultured under normoxic conditions (Fig. 6D). This hypothesis is supported by the demonstration that overexpression of miR10b in parental SK-BR3 cells resulted in increased MT1-MMP expression. Conversely, silencing of miR10b expression in SK-3rd TICs significantly reduced MT1-MMP expression. Our results provide experimental evidence to support hypotheses proposed in recent reports [30], [31], [39].

Hypoxia has been reported to induce MT1-MMP expression in retinal glial cells and hepatocellular carcinoma cells using a standard RT-PCR approach [40], [41]. However, using real time quantitative RT-PCR, we demonstrate that hypoxia does not induce MT1-MMP mRNA and protein expression in breast cancer TICs. Instead, hypoxia results in trafficking of preexisting MT1-MMP in SK-3rd TICs from cytoplasmic storage pools to cell surface, which is in agreement with a previous report showing that hypoxia induced MT1-MMP cell surface translocation in MDA-MB-231 cells [42]. Our data showing that treatment of parental SK-BR3 cells with CoCl_2_ did not enhance cell invasion is supportive of our observation that MT1-MMP expression is not regulated by hypoxia. Storage of MT1-MMP in cytoplasmic storage pools has been previously reported in other cancer cell lines [29]. Upon stimulation, MT1-MMP relocates from the storage pool to the cell surface possibly via Rab8 [43] and VAMP7 [44] pathways. Based on other reports, it is suggested that hypoxia promotes RhoA-dependent trafficking of MT1-MMP to the plasma membrane [42]. Furthermore, we have recently shown a role for the chaperone protein, Hsp90α, in trafficking of MT1-MMP to the plasma membrane (manuscript in preparation).

Currently, isolation of cancer stem-like cells has been mainly based on cell surface marker and tumor-sphere formation. Indeed, in addition to tumorigenic capability, expression of cell surface CD44 and the ability to form tumor-spheres are two intrinsic markers of cancer stem-like cells [45]. However, we observed that culture of SK-3rd TICs or colon TICs under hypoxic conditions resulted in a gradual increase in cell surface MT1-MMP and decrease in cell surface CD44 expression, as examined by flow cytometry, cell surface biotinylation, and cellular functional assays for CD44 and MT1-MMP (Fig. 2F & G). Furthermore, increased MT1-MMP with diminished CD44 at the cell surface of SK-3rd TICs results in dissociation of mammospheres (Fig. 3E). These observations are consistent with the concept that the plasticity of TICs undergoes dynamic changes at different stages of metastasis. In a separate study, we observed the reappearance of E-cadherin, a cell-cell junction adhesion molecule, in MT1-MMP transfected LNCaP prostate tumors at metastatic sites in the lungs (high oxygen content) compared with the primary tumor in samples from an orthotopic prostate cancer model (manuscript in preparation). Since the current procedure for isolation of TICs from human cancer specimens is commonly based on cell surface markers and the ability to form mammospheres, our data suggest that invading/metastasizing TICs may not be identified using these criteria due to a dynamic change in CD44 and mammosphere-forming ability. The issue of whether the loss of mammosphere formation and decrease of cell surface CD44 by hypoxia-upregulated MT1-MMP in SK-3rd cells excludes these cells from being considered stem-like cells is a semantic one, since plasticity is a recognized aspect of stem-like cells and we demonstrated that four days after return to normoxia, partial restoration of the stem-like cell phenotype of SK-3rd TICs occurs.

Mesenchymal cells need to be reconverted to an epithelial phenotype (MET) in order for TICs to proliferate at distant metastatic sites [46]. Hypoxia-induced MT1-MMP appearance on the cell surface persists for four days after returning to normoxia (Fig. 3F). This time period is consistent with the slow extravasation of cells from the vasculature during breast cancer micrometastasis [47]. In addition, when hypoxia-cultured TICs returned to normoxic conditions for a longer time period, cell surface MT1-MMP gradually disappeared. Conversely, cell surface CD44 reappeared (Fig. 3F). These observations support phenotypic transition in response to changes in the microenvironment, referred to as EMT and MET conversion. Due to technical limitations in capturing the critical role of MT1-MMP cell surface trafficking during EMT and MET dynamic changes *in vivo*, monitoring MT1-MMP turnover using the hypoxia-normoxia *in* *vitro* switching model will help us to better understand this important aspect of metastasis.

Of relevance to our studies, Biddle *et al*. [48] have isolated a TIC subpopulation from human oropharyngeal cancer cells that have undergone EMT and are migratory. The EMT (sphere-forming, migratory, and metastatic *in vivo*) and non-EMT (rapid proliferation as adherent and less metastatic cells) TIC populations coexist by switching between their phenotypes. They proposed that TICs have the ability to take on an EMT phenotype for migration to a secondary site, before reverting back to the proliferative non-EMT phenotype to enable formation of a metastatic tumor at that secondary site. This experimental EMT-MET conversion is consistent with the theory proposed by Brabletz *et al* [8]. Our studies describing the role of hypoxic-induction of MT1-MMP trafficking to the cell surface as being of central importance in the conversion of stationary to invasive TICs provides a mechanistic explanation for EMT-MET in cancer metastasis.

In conclusion, we have demonstrated for the first time that hypoxia induces the conversion of less invasive and stationary TICs to become invasive and metastatic by promoting MT1-MMP cell surface trafficking. Effect of oxygen tension on plasticity of TICs regulates metastatic process via MT1-MMP cell surface appearance and disappearance. Hence, targeting MT1-MMP represents a viable approach for preventing cancer dissemination.

## Materials and Methods

### Reagents

Type 1 collagen (acetic acid-extracted native type 1 collagen from rat-tail tendon) was purchased from BD Bioscience Discovery Labware (Bedford, MA). EZ-link Sulfo-NHS-S-S-Biotin was purchased from Pierce. RNAi-Ready pSIREN-RetroQ and GFP vectors were purchased from Clontech (Mountain View, CA). Plasmid DNAs including MDH1-PGK-GFP 2.0 [49], MDH1-PGK-GFP miR10b [30] and pBabe-puro-miR10b sponge [50] were purchased from Addgene (**Cambridge, MA)**. Primary antibodies were purchased from Abcam (Cambridge, MA; anti-MT1-MMP hinge polyclonal antibody), Cell Signaling Technology (Beverly, MA, anti-β-actin and anti-α,β-tubulin antibodies), Santa Cruz (Santa Cruz, CA, anti-vimentin, anti-cytokeratin8/18, anti-HCAM (CD44) and anti-HoxD10 antibodies), BD Biosciences (San Jose, CA, anti-HIF-1αantibody), and Zymed Laboratories Inc (Carlsbad, CA, anti-E-cadherin antibody against extracellular portion). Secondary antibodies (anti-Mouse and anti-Rabbit IgG) were purchased from Rockland Immunochemicals (Gilbertsville, PA). Recombinant pro-MMP-2 was produced in COS-1 cells transfected with MMP-2 cDNA [51].

### Cell Culture

The SK-3rd cells were maintained as previously described [14]. The SK-3rd cells were cultured in ultra-low attachment culture dishes (Corning) in suspension with serum-free DMEM-F12 (Cellgro) medium supplemented with B27 (1∶50, Invitrogen), 20 ng/mL EGF (BD Biosciences), 0.4% bovine serum albumin (Sigma), and 4 µg/mL insulin (Sigma) [52]. SK-BR3 cells were cultured in McCoy’s 5A medium (Gibco) supplemented with fetal bovine serum (Invitrogen) to a final concentration of 10%. Both SK-3rd and SK-BR3 cells were cultured under a 5% CO_2_ atmosphere.

### Mammosphere Culture

Mammosphere culture was performed as previously described [52]with a slight modification. Cells (1000 cells/ml) were cultured in ultra-low attachment culture dishes in suspension for 12 days. For sphere passage *in vitro*, spheres were collected by gentle centrifugation, dissociated to single cells, and then cultured to generate mammospheres for the next generation.

### Flow Cytometry Analysis

APC-conjugated anti-CD44 (Clone G44-26) antibody, PE-conjugated anti-CD24 antibody (clone ML5) and propidium iodide (PI) (10 µg/mL) were purchased from BD Biosciences and used for flow cytometry analysis in accordance with the manufacturer’s protocols. 1×10^6^ cells/ml suspended in PBS containing 2% BSA were incubated with CD44/CD24 conjugated antibodies or anti-MT1-MMP hinge polyclonal antibody for 60 min at 4°C followed by incubation with anti-rabbit FITC-labeled (1∶1000) secondary antibody for 60 min at 4°C (for MT1-MMP staining). After extensive washing, the cells were stained with PI to determine viability of the cells. Cell surface CD44, CD24, and MT1-MMP expression was measured using a FACS Calibur flow cytometer (BD Biosciences). Only the PI negative cell population was gated for further analysis.

### RNA Isolation and Real Time Reverse Transcription-PCR

Total RNAs were isolated from cultured cells using a RNA Purification kit (QIAGEN, Valencia, CA). Reverse transcription was performed using a Bio-Rad iScript cDNA synthesis kit based on product instructions. The resultant cDNAs were used for PCR using iQ SYBR Green Supermix (Bio-Rad, Hercules, CA) in triplicates. PCR and data collection were performed on MyIQ2 real time PCR system (Bio-Rad). The relative quantitation value for each target gene compared with the endogenous controls (GAPDH and HPRT) for that target was analyzed by a ΔΔCT method (MyIQ2 software; Bio-Rad).

The primer sets used for real time RT-PCR were as follows: MT1-MMP (NM_004995), sense (cactgcctacgagaggaagg) and anti-sense (ttggggtactcgctatccac); cytokeratin-8 (M342250), sense (ccgacgagatcaacttcctc) and anti-sense (ggctctgcagctcctcatac); cytokeratin-18 (X12881), sense (aaaggcctacaagcccagat) and anti-sense (cactgtggtgctctcctcaa); vimentin (NM_003380), sense (ccctcacctgtgaagtggat) and anti-sense (tccagcagcttcctgtaggt); GAPDH (NM_002046), sense (ctcatgaccacagtccatgc) and anti-sense (tttctagacggcaggtcagg); HPRT1 (NM_000194), sense (accccacgaagtgttggata) and anti-sense (aagcagatggccacagaact); Snail (NM_005985), sense (gcgagctgcaggactctaat) and anti-sense (cccactgtcctcatctgaca) andTwist1 (NM_000474), sense (gtccgcagtcttacgaggag) and anti-sense (ccagcttgagggtctgaatc).

For microRNA (miRNA) analysis, total RNAs, including miRNAs, were isolated from the cell lines using TRIzol reagent (Invitrogen Corporation) in accordance with the manufacturer’s instructions. Ten nanograms of total RNAs from each cell line were converted to cDNA using the High-Capacity cDNA synthesis kit (Applied Biosystems). The miRNA sequence-specific reverse transcription primers for miR-10b and an endogenous control RNU6B were purchased from Ambion (Austin, TX, USA). Real-time quantitative RT-PCR analysis was performed using the 7500 Real-Time PCR System (Applied Biosystems). The relative quantification value was calculated using the comparative *C*_T_ (ΔΔ*C*_T_) method (Applied Biosystems) and normalized by the expression of the internal control RNU6B. Three independent experiments were performed.

### Hyaluronic Acid Adhesion Assay

96-well tissue culture plates were coated with 3 mg/ml hyaluronic acid in PBS or 2% BSA as a control overnight at 4°C. Nonspecific sites were blocked with 2% BSA for 10 minutes at room temperature. The wells were washed with PBS and seeded with cells (60,000 cells/well) previously incubated with 2.5 µM calcein in phenol-free medium. Adhesion was allowed to proceed at 37°C for 1 h. The wells were then washed two times with PBS, and plate was read on a Molecular Devices Gemini EM (excitation 494, emission 517).

### Animal Experiments

The tumorigenicity study was performed by **orthotopic injection** of tumor cells with 50% Matrigel into the mammary fat pad of NCR-Nu-M female mice (4–5 wks old) (Taconic). Tumor incidence was monitored and tumor size was measured twice per week. After four weeks, the mice were sacrificed and primary tumors were resected. Major organs were also harvested and examined for tumor metastasis based on GFP autofluorescence.

### Statistical Analysis

Data are expressed as the mean ± S.E. of triplicates. Each in vitro experiment was repeated as least 3 times. Student’s *t* test and analysis of variants were used to assess differences with *p*<0.05 considered to be significant.

### Construction of Small Interference RNA Vectors and Retroviral Infection, Gelatin Substrate Zymography, Cell Surface Biotinylation, MTT proliferation assay, and Western Blot analysis

Basic protocols for above techniques have been described previously [12], [13].
